# Juvenile Dermatomyositis Presenting As Generalized Poikiloderma: A Case Report

**DOI:** 10.7759/cureus.50573

**Published:** 2023-12-15

**Authors:** Sarah Alaboud, Wafi Al Hawsawi, Nouf Alqahtani, Mohammad Aldosari, Waseem K Alhawsawi, Khalid Al Hawsawi

**Affiliations:** 1 College of Medicine, Umm Al-Qura University, Makkah, SAU; 2 Collage of Medicine, King Saud Bin Abdulaziz University for Health Sciences, Jeddah, SAU; 3 Dermatology, King Abdulaziz Hospital, Makkah, SAU; 4 Pathology, King Abdulaziz Hospital, Makkah, SAU; 5 Dermatology, King Fahad Armed Forces Hospital, Jeddah, SAU

**Keywords:** poikiloderma, secondary icthyiosis, immune-mediated myopathy, chronic autoimmune disease, juvenile dermatomysitis

## Abstract

Juvenile dermatomyositis (JDM) is a chronic autoimmune inflammatory disorder and is considered the most common form of idiopathic inflammatory myopathies. JDM primarily affects the skin and the skeletal muscles. Characteristic signs and symptoms include Gottron papules, heliotrope rash, calcinosis cutis, and symmetrical proximal muscle weakness. However, JDM presenting with generalized scaly poikeloderma is an unfamiliar presentation. Herein we report a 14-month-old female toddler presented with generalized progressive asymptomatic scaly mottled violaceous patches (poikilodermatous) that started when she was seven months old. Her lab results were unremarkable. She was diagnosed with poikilodermatous skin rash with a differential diagnosis of Amyopathic dermatomyositis, poikilodermatous genodermatosis, and patch-stage mycosis fungoides. She was prescribed moisturizer creams only. A year later, during a follow-up, she presented with a full picture of JDM, with a history of scaly poikilodermatous skin patches that became more widespread, frequent choking during oral intake, and not being able to stand and sit unsupported. Laboratory workup was significant for low WBC and hemoglobin counts, along with elevated CPK, LDH, ferritin, CRP, and ESR levels. MRI revealed the right anterior thigh and vastus lateralis subcutaneous edema. Therefore, the child was diagnosed and treated as a case of JDM.

## Introduction

Dermatomyositis is an autoimmune connective tissue disorder of unknown etiology showing bimodal age distribution; Juvenile dermatomyositis (JDM) and adult form dermatomyositis. In the absence of cutaneous changes, the term polymyositis is used [1]. Cutaneous manifestations are variable, including Heliotrope rash, Gottron’s papules/sign, nailfold capillary changes, facial malar eruption, mouth/skin ulcers, gingival telangiectasia, limb edema, xerosis, poikiloderma, calcinosis, and lipodystrophy. Non-specific constitutional symptoms such as fever, lethargy, and adenopathy can present in JDM cases. Dyspnea should raise the suspicion of interstitial lung disease, and rarely cardiac involvement. The diagnosis can be established through the score-based EULAR/ACR classification criteria. Criteria elements represent the aforementioned classical clinical features, in addition to the age of onset, elevated muscle-derived serum enzyme levels, muscle biopsy/MRI, and myositis-specific antibodies.1 JDM is treated with systemic steroids, methotrexate, and/or cyclosporin in mild to moderate disease. Intravenous immune globulin is used in refractory or recurrent disease [2-4]. Several other immunomodulators including rituximab, anti-TNF agents, JAK-STAT inhibitors, and many other agents are under investigation and show promising results [5-7]. JDM presenting with generalized scaly poikeloderma is an unfamiliar presentation. It is important to encourage clinicians to share their experience in JDM atypical presentations to reach the goal of easier and earlier detection of the disease.

## Case presentation

**Figure 1:**
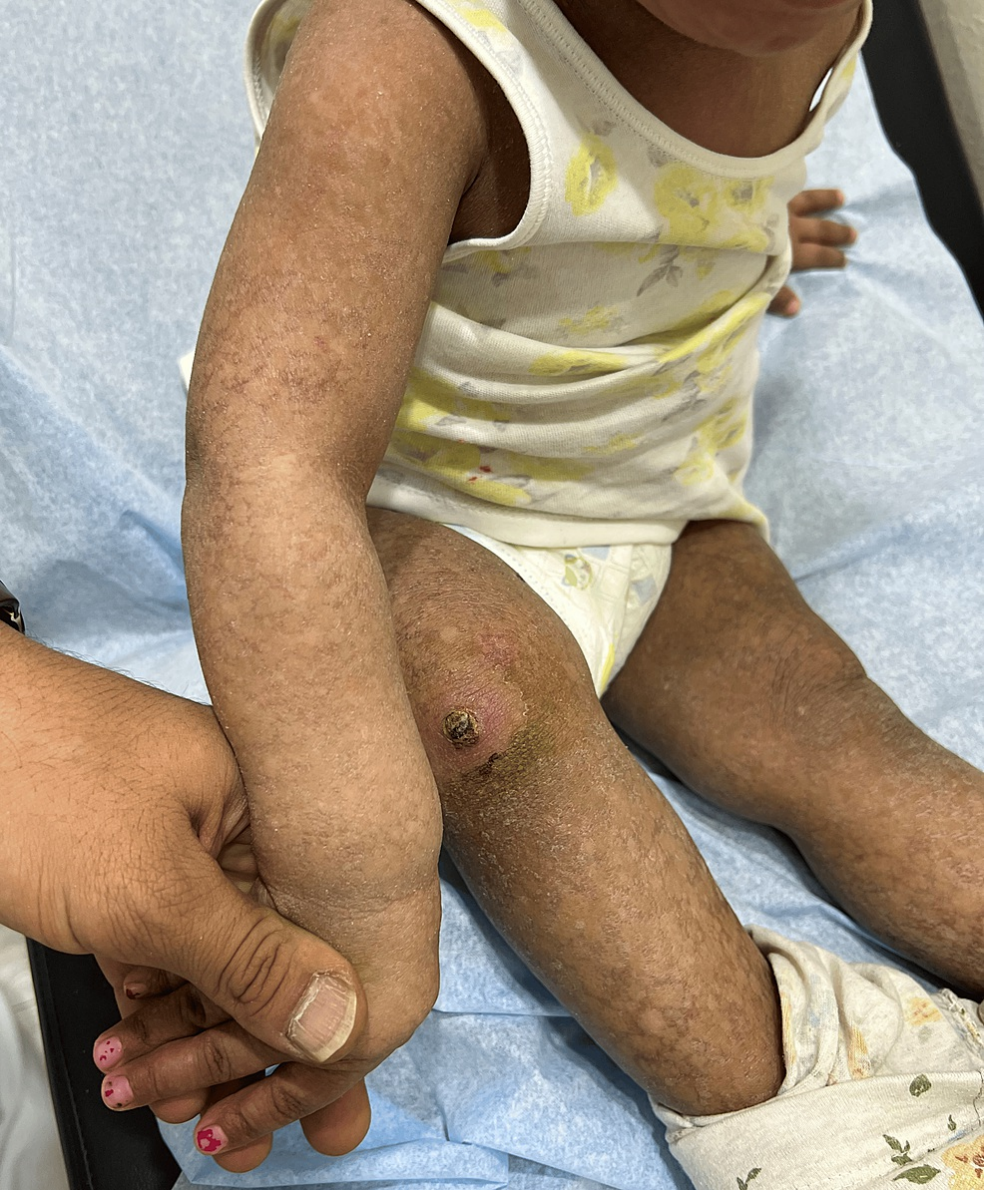
Generalized poikiloderma affecting all extremities.

**Figure 2:**
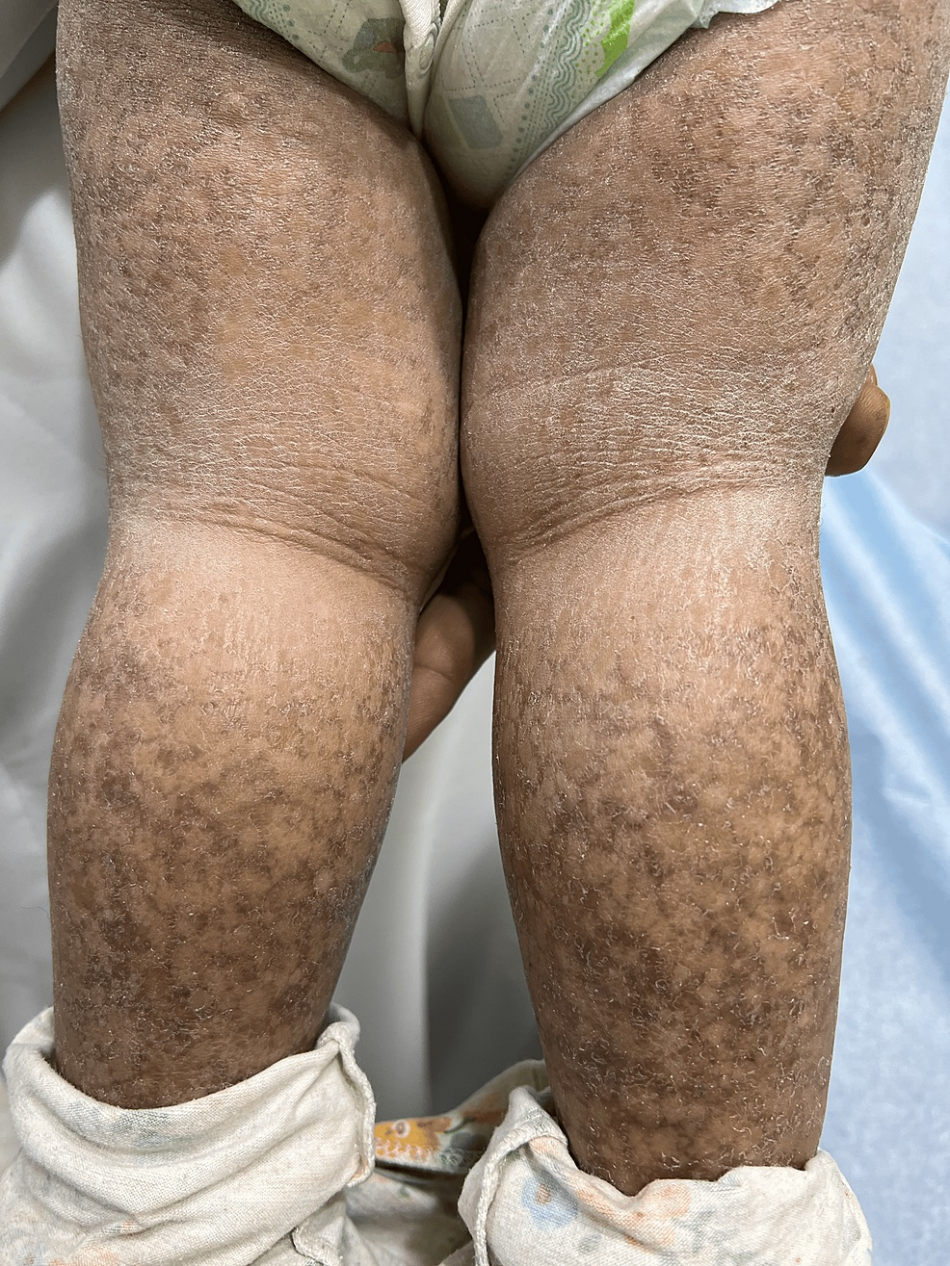
Closer view of poikiloderma.

A 14-month-old female toddler, not known to have medical illnesses, presented to our clinic with generalized progressive asymptomatic skin lesions that started when she was seven months old. Her perinatal history was uneventful. Systemic review did not reveal a change in the child's activity. No history of (H/O) frequent choking during oral intake. No H/O irritability. No H/O fever. Family history was unremarkable and there was no similar case in the family. Developmental milestones were reached for her age. Skin examination revealed multiple scaly mottled violaceous patches on her upper and lower extremities (Figures 1, 2).

**Figure 3:**
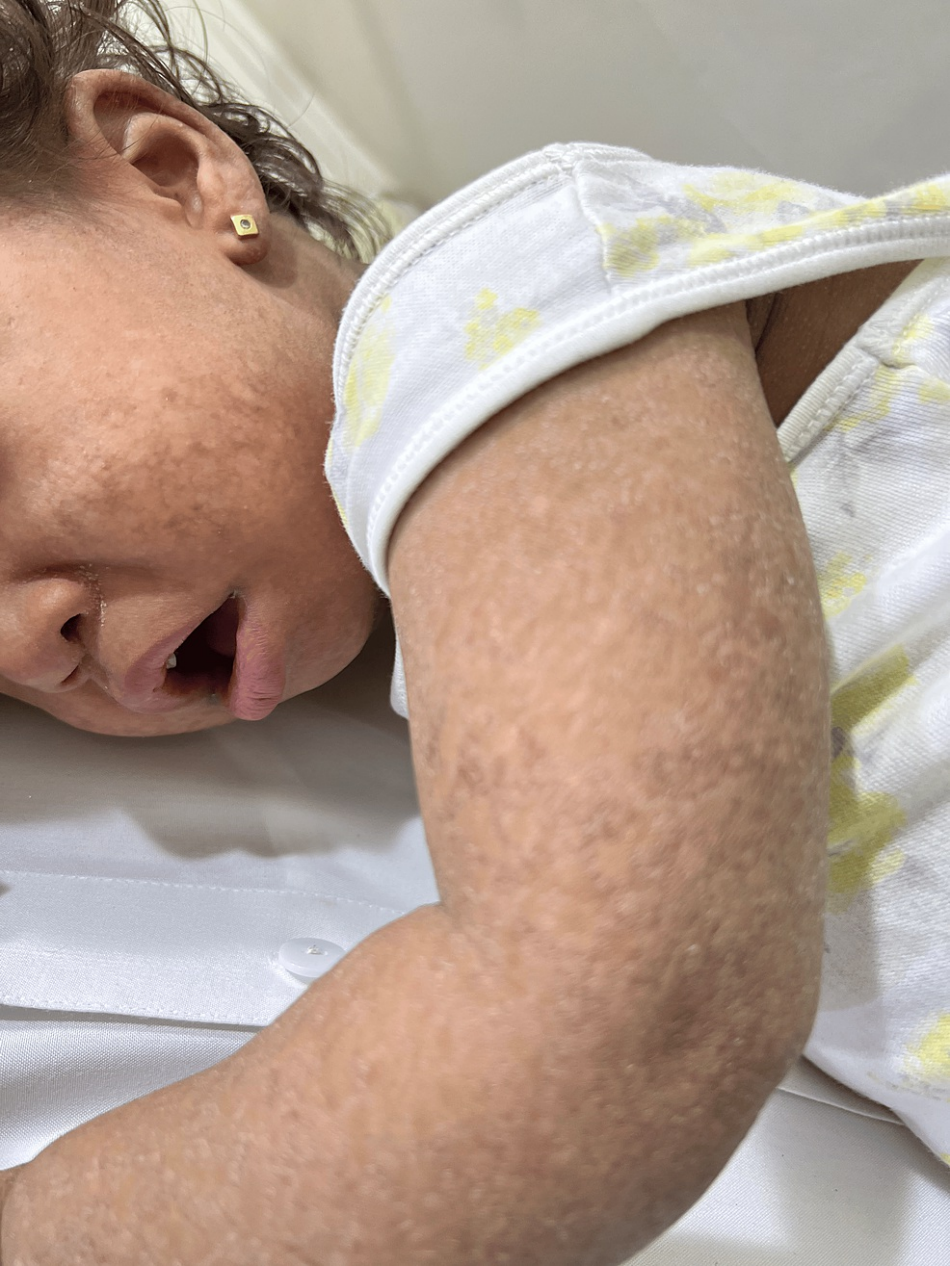
Poikiloderma with facial involvement.

**Figure 4:**
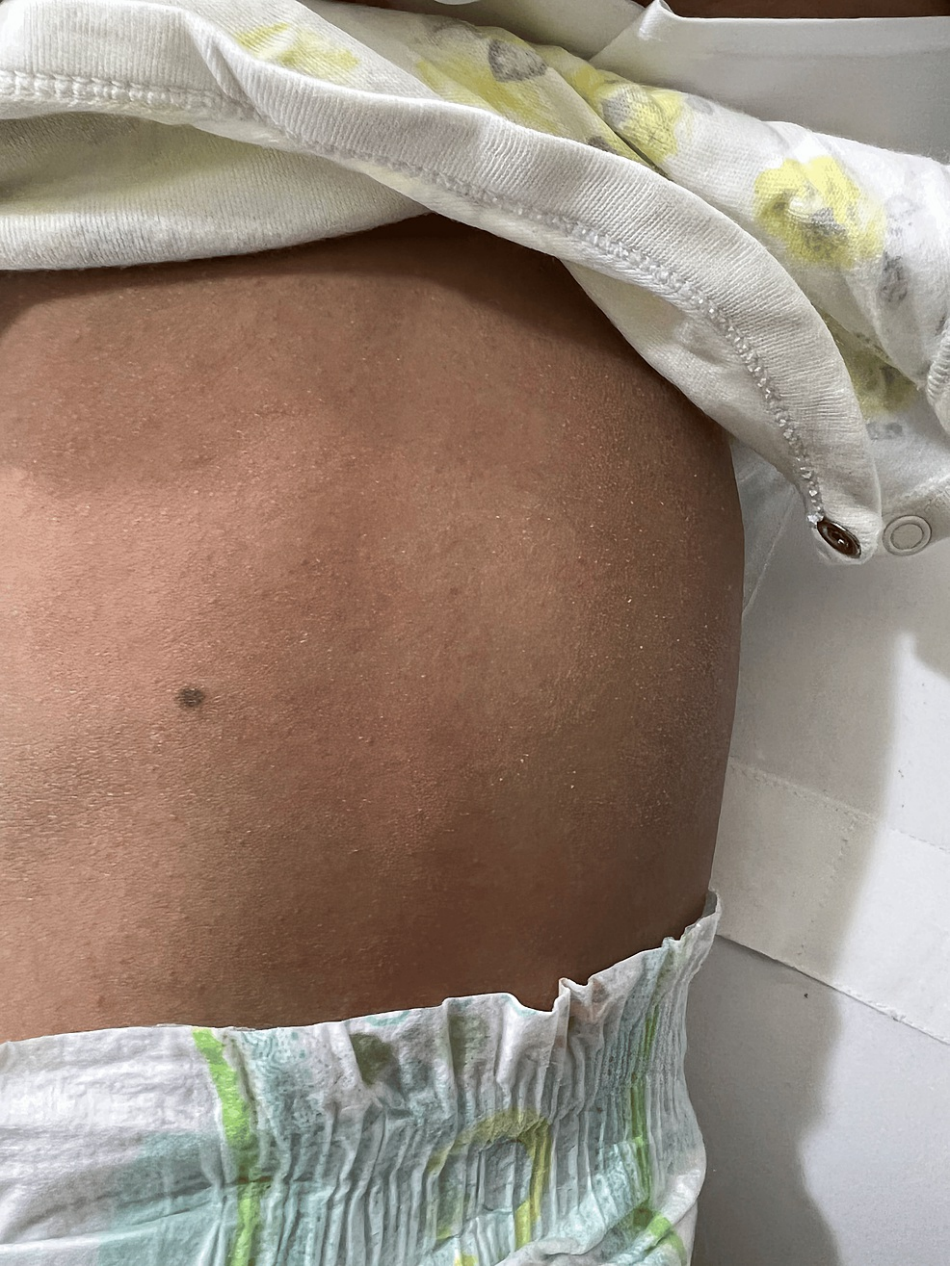
Trunk showing scaly erythema with subtle poikiloderma.

Lesional skin punch biopsy showed mild perivascular dermal lymphocytic infiltration with melanin inconvenience. Laboratory workup was insignificant for CBC, CPK, LDH, AST, ferritin, CRP, and ESR levels. ANA and dsDNA were negative. Based on the above clinicopathological findings, the baby was diagnosed with poikilodermatous skin rash with a differential diagnosis of amyopathic dermatomyositis, poikilodermatous genodermatosis, and patch-stage mycosis fungoides. She was prescribed moisturizer creams only. A year later, during follow-up, at the age of 26 months, the scaly poikilodermatous skin patches became more widespread. The mother stated that she is not happy with her child's activity. The baby has a history of frequent choking during oral intake. She is also not able to stand and prefers always to be carried up. Mother described her to be always unhappy and irritable. The baby still cannot sit unsupported, nor can she bear her weight while standing. Developmental milestones, other than gross motor delay, were reached for her age. Skin examination revealed generalized scaly poikilodermatous patches all over her body with mild involvement of the trunk and face (Figures 3, 4).

Laboratory workup was significant for low WBC and hemoglobin counts, along with elevated CPK, LDH, ferritin, CRP, and ESR levels. ANA and dsDNA were negative. MRI revealed the right anterior thigh and vastus lateralis subcutaneous edema. Pan CT scan did not show any hidden masses. Given the aforementioned information, the child was labeled as JDM. The child was admitted under rheumatology and received three doses of pulse methylprednisolone and IVIG 2 g/kg. The baby was put on prednisolone syp 1 mg/kg once daily and methotrexate SC injection 1 mg/kg once weekly as maintenance therapy with excellent responses.

## Discussion

Although both dermatomyositis and polymyositis are considered a spectrum of the same disease entity, the pathophysiology of tissue destruction was discovered recently to be different. Muscle fiber degeneration and necrosis are mediated by cytotoxic T-cells, whereas cutaneous changes are caused by humoral antibody- and complement-mediated capillary vasculopathy. The peak incidence is during school ages and girls are affected two- to fivefold greater rate than boys. However, our patient showed onset during the infantile period. The hallmark of JDM is symmetrical proximal extensor muscle weakness, usually with myalgia. Involvement of palatal and cricopharyngeal muscles is common, causing problems while swallowing. JDM can present with many distinct cutaneous features. Heliotrope sign and Gottron’s papules are classically considered pathognomic for the disease, however, both were absent in our patient. Poikiloderma can affect both photodistributed and photoprotected areas, the former is very characteristic for dermatomyositis. However, our patient showed generalized scaly poikiloderma, a feature that is rarely seen in DM. Such atypical presentations can delay the diagnosis and prevent patients from the benefit of early treatment of such debilitating diseases, especially in this age group.

Amyopathic JDM is very rare in the pediatric age group accounting for about 5% of JDM cases [1]. In one series included 166 newly diagnosed children with JDM showed that skin rash is the presenting symptom in 65% of cases [2]. Our case was initially diagnosed as amyopathic DM; however, six months later she developed clinical, laboratory, and imaging features of JDM. So, one should not hurry to label the case as amyopathic JDM until two years from the onset of the disease, where after that time a definitive diagnosis of amyopathic JDM can be made if the patient did not develop muscle weakness [3].

Unlike adult DM, children do not have an increased risk of internal malignancy, so no workup of internal malignancy was ordered. To our knowledge, our case is the first case in the literature showing JDM with generalized poikiloderma.

## Conclusions

JDM cutaneous features are variable but rarely present with generalized poikeloderma. It is important to encourage clinicians to share their experience in JDM atypical presentations to reach the goal of easier and earlier detection of the disease. A definitive diagnosis of amyopathic JDM is made if the patient does not develop muscle weakness for two years after the onset of the skin rash.
